# Parents' Initial Perceptions of Multidisciplinary Care for Pediatric Chronic Pain

**DOI:** 10.1155/2012/791061

**Published:** 2012-08-21

**Authors:** Ayala Y. Gorodzinsky, Susan T. Tran, Gustavo R. Medrano, Katie M. Fleischman, Kimberly J. Anderson-Khan, Renee J. Ladwig, Steven J. Weisman

**Affiliations:** ^1^Department of Psychology, University of Wisconsin-Milwaukee, 2441 East Hartford Avenue, Milwaukee, WI 53211, USA; ^2^Medical College of Wisconsin, 8701 Watertown Plank Road, Milwaukee, WI 53226, USA; ^3^Children's Hospital of Wisconsin, 9000 West Wisconsin Avenue, Milwaukee, WI 53226, USA

## Abstract

Chronic and recurrent pain is experienced by many children and adolescents. Treatment of chronic pain using a multidisciplinary approach has been found to be effective for treatment of chronic pain. Parent satisfaction with treatment and treatment providers highly correlates to children's treatment adherence. Parents of children treated at a multidisciplinary chronic pain clinic were interviewed following their initial appointment. Parents reported high satisfaction with treatment team members and with the treatment plan. Parents also reported appreciation of multidisciplinary structure, the high level of expertise of the team members, and the team members' genuine interest in treating their children. This increase in satisfaction when compared to previous treatment is important since increases in satisfaction may correlate with a reduction in experiences of chronic pain. Parents reported high satisfaction with interactions with treatment team members and with the treatment plan provided for their children. Parents had appreciation of multidisciplinary team structure and the high level of expertise of the team members. This increase in satisfaction when compared to treatment from previous providers is important since increases in satisfaction may correlate with an increase in children's treatment adherence and a reduction in experiences of chronic pain.

## 1. Introduction

Chronic, persistent, and episodic pain has a prevalence rate of 25 to 33% in childhood and adolescence [1]. Treatment of chronic pain is essential as chronic pain is disruptive both to the functioning and development of the individual experiencing pain [2]. Children with chronic pain experience significant interference with various developmental functioning, such as attending school [3] and increased levels of emotional distress and impairment [4]. In an effort to find treatment for chronic pain, patients must often negotiate appointments with multiple providers, including both primary (e.g., pediatrician) and secondary (e.g., neurologist) providers [5]. Consequences of searching for treatment often includes missed school for child, missed work for parents, and use of emotional and financial resources [6].

Families often report limited belief by health care providers regarding the high levels of pain their child is experiencing, which can interfere with the trust and relationship the families create with the providers. Parents may experience a general distrust of the medical system and frustration with a lack of conclusive medical tests [7], decreasing their hopes that any health provider will be able to do anything for their child. Due to the failure of treatments to reduce their children's pain, parents may experience reduced expectations for treatment [8].

Since chronic pain is not solely a response to physical sensations, a biopsychosocial approach to treatment is beneficial [9]. Despite few parents and children reporting use or consideration of psychological treatments for pain [10], researchers have found that a multidisciplinary approach implementing a biopsychosocial perspective to assess and treat chronic pain in youth is effective in reducing the pain experiences and consequences of pain [5, 9–11]. Treatment of chronic pain should address use of medications, physical treatment, and psychological treatment [12, 13].

The use of a multidisciplinary approach to treatment has been found to be more acceptable to parents and patients than a focus only on the medical factors of pain, and families note that providers in these multidisciplinary settings displayed a noted interest in understanding the pain and increasing the comfort of the families during the appointment [14]. This focus on integrating families' perspectives and increasing family members' comfort during treatment is captured by the concept of collaborative health care. Collaborative health care focuses on utilizing the patient's and family's perspective of prognosis and treatment into the planning of treatment [15]. This orientation is vital for increased treatment adherence since patients and families need to be invested and cooperative with the treatment plan in order to implement it effectively [15, 16]. As well, collaboration between health care professionals during treatment planning can provide patients and families with a better understanding of the treatment plan and increase effective communication regarding the treatment plan.

The current literature provides information regarding the importance of using the biopsychosocial perspective for treating patients with chronic pain; however, it is currently unclear how parents of patients with chronic pain perceive the multidisciplinary team approach to treatment using the biopsychosocial perspective. In the current literature, there is some information regarding parents' perceptions of treatment provided using the biopsychosocial perspective; however, this information is not based on patients with various types of chronic pain. The current study assesses treatment provided at a multidisciplinary chronic pain clinic treating various types of chronic pain, such as headache, abdominal pain, and musculoskeletal pain. The current study adds to the literature an understanding of parents' perceptions of the multidisciplinary team approach to treatment of their children's chronic pain.

## 2. Methods

The multidisciplinary clinic team includes a pediatric anesthesiologist with specialization in pediatric pain, a licensed clinical psychologist, a licensed marriage and family therapist, and physical and occupational therapists. Each year, this multidisciplinary clinic has an average of 280 intake appointments for children with various chronic pain concerns. Patients are typically referred to this clinic by their primary care physician or by a specialist after other medical services provided have failed to alleviate their pain experiences or testing has resulted in no organic etiology of pain experience.

For all initial pain clinic evaluations, the patient and parent(s) meet with a physician and psychologist or family therapist for a 1.5 hour visit. The family is interviewed jointly for the medical history and general functioning information. Then while the physician and, if appropriate, physical therapist conduct a physical assessment of the patient, the mental health provider interviews the parent(s). After the physical exam is completed, the mental health provider interviews the patient. The physician and mental health provider then meet briefly to draft a treatment plan, which is then presented to the family. The multidisciplinary team intervention during this visit includes careful attention to medical and illness narratives and family beliefs and expectations, validation and acknowledgement of challenges and strengths, development of a shared rationale for understanding patient and parent distress, testing parental and patient receptiveness to initial reframes of maladaptive beliefs, provision of a rationale for treatment recommendations, and development of a comprehensive treatment plan. Treatment plan recommendations are individually tailored and may include medication management, physical therapy, individual and family cognitive behavioral therapy, school accommodation and return to school plan, collaboration with existing medical and mental health providers and school staff, and pain team followup.

Parents were asked to share their experiences and perspectives of the initial intake appointment. Questions included their initial expectations of the intake appointment and treatment plan, how these expectations changed during and after the appointment, and their perceptions of the structure of the multidisciplinary nature of the intake appointment (see Appendix). The initial appointment at the multidisciplinary clinic is family-centered treatment and structured for collaboration not only between the multiple health providers but also between the providers and the family members. In the initial appointment, the health providers strive to increase families' understanding of the pain within the biopsychosocial model, to increase families' trust in the team, to provide the families with a comprehensive treatment plan, and to increase families' hope in restoring functioning and possibly reducing youths' pain experiences.

Data was collected from nine parents of adolescents that were seen as outpatients at a pediatric pain management clinic in a large mid-western city for the treatment of their chronic pain. During the initial appointment, members of the multidisciplinary pain treatment team gave a brief written description of the study to the parents. Parents who were interested signed consent forms and an appointment was scheduled for the interview to be conducted in the family's home within one week of the initial intake with the multidisciplinary team. This study was reviewed and approved by the university's and local hospital's IRB committee. No parents were excluded from the study based on gender, race, or ethnicity.

This study utilized a qualitative design based on Consensual Qualitative Research (CQR^17^). Based on the CQR methodology, we explored and described in depth parents' expectations and experiences during an intake encounter with a multidisciplinary pain team. Our study used an open-ended interview to capture the parent's experience in order to describe the phenomena as it naturally occurred. No specific hypotheses were developed; instead, inductive analysis was used to draw conclusions from the data obtained by the parent's responses. CQR is a replicable method that instills scientific rigor by using multiple researchers that form a consensus on decisions and verify results. Furthermore, using multiple researchers helps minimize bias by sharing a variety of opinions and perspectives that capture the data [17]. Within the framework of CQR, acceptable sample sizes range from 8 to 12 participants [17].

Providers at the multidisciplinary clinic, in collaboration with researchers from the counseling and clinical psychology program, developed the interview protocol based on clinical experience and literature related to multidisciplinary pain treatment and adolescent chronic pain. The interview guide included open-ended topics such as expectations and experiences prior to the clinic appointment, description of thoughts and feelings about the appointment, and treatment recommendations (see Appendix).

Members from the counseling and clinical psychology research teams and researchers from the medical college completed nine interviews. The interviews took place at the patient's residence to ensure comfort and confidentiality. All questions were asked to each parent and lists of probes were used to gather any additional information or clarification. Furthermore, additional probes were used to explore issues that emerged in the interview for each participant [17]. All interviews were audio recorded and then transcribed verbatim. As soon as the transcripts were completed and deidentified, all audiotapes were destroyed.

The primary coding team consisted of three clinical psychology doctoral students, who were not involved in interviewing participants. All members read articles [17, 18] and held several discussions on CQR and qualitative interviewing. A counseling psychology doctoral student and counseling psychology faculty member who are experienced in CQR methodology served as the coauditors. Before the data was collected, all members discussed their expectations and biases. A discussion of these expectations and biases continued throughout the remainder of the study to ensure results were derived from the data.

There were several steps in the procedures for analyzing the data. First, domains (i.e., themes) were constructed by dividing up the responses to the open-ended interview questions. Second, core ideas (i.e., summaries) were formed from all the material from each domain for each parent's response. Third, cross-analysis was performed by finding common themes transcending the domains and core ideas. Along the process, the primary team made judgments about each decision until consensus was reached and the best structure developed. At each step, the raw data was continually reviewed making sure the final decisions were based on the data. Lastly, the auditor viewed the judgments during the cross analysis stage to ensure the primary team did not fail noticing any of the essential data [19]. That is, the role of the auditor involved independently checking on the accuracy of interpretations at each stage of the analysis (e.g., reviews the domain themes, examines whether raw data is in the correct domain). The auditor could suggest combining, changing, or deleting at each stage to make sure all data is represented. The coding team received the auditor's comments and could accept or reject any or all of them.

## 3. Results

The purpose of this study was to examine parents' thoughts and feelings about their child's chronic pain, past treatment, and initial evaluation by a multidisciplinary chronic pain team. Overall, 14 domains were identified based on the content across all nine interviews. Within these domains, 85 core ideas were determined to exist across interviews. According to the procedure described by Hill and colleagues [18], categories were developed from core ideas across all parents within each domain. Core ideas were categorized as “general” if the idea appeared in eight or nine interviews, “typical” if it appeared in four to seven interviews, and “variant” if it appeared in two or three interviews. Core ideas that only appeared in one interview were categorized as “rare” and were not included for further analysis. The nine domains regarding information about the parent's perceptions of care at the multidisciplinary clinic are described below (Patient's Prior Treatment for Chronic Pain, Parent-Past Provider Relationship, Parent Attitudes toward the Experience and Treatment of Pain, Parent Preconceptions of Appointment, Parent Attitudes toward Multidisciplinary Team, Child Attitudes toward Multidisciplinary Team, Parent-Clinic Provider, Patient's Noncompliance and Treatment Plan); the other five domains included background information about the child and his or her pain and are not included for analysis in this paper (Pain History, Child's Social Experiences, School Experiences, Parent-Child Relationship, and Clinic Logistics) as these domains did not address the specific interest of how families perceive their treatment at the multidisciplinary clinic (details of these domains are available upon request).

### 3.1. Parent's Perception of Prior Treatment for and Experiences with Chronic Pain

#### 3.1.1. Patient's Prior Treatment for Chronic Pain

The domain Patient's Prior Treatment for Chronic Pain contains objective descriptions of the child's past treatments for pain. This domain includes four typical (Doctors/Providers, Hospitals, Medication, and Treatment Novelty) and two variant (Surgery and Team Novelty) core ideas. Parent reports typically described seeing a variety of medical providers for their children's pain (e.g., “*We've been from doctor… to doctor and we still do not really have a diagnosis*”), having been seen in a hospital for pediatric pain, and using medication to manage pediatric pain. Parents also typically discussed never having had a treatment plan in place for the child's pain before coming to the pain clinic. Variant reports include information about the child having undergone surgery for pain and statements about the child never having met with a team of providers before to treat their pain (e.g., “*Well, that was our first time we met with a pain team you know, a pain clinic…*”).

#### 3.1.2. Parent-Past Provider Relationship

The domain Parent-Past Provider Relationship describes parents' contact and rapport with past medical doctors. This domain includes five typical (Hospital System, Negative Interactions, Positive Interactions, Short Visits, and Treatment Disagreement) and four variant (Disbelief, Financial System, Lack of Expertise, and Talking without Child) core ideas. Typically, parents discussed a general lack of centralization of providers at many hospitals, their general problems with hospital systems, seeing providers for only short amounts of time at their appointments (e.g., “*You almost felt like they had to get you in and out in ten minutes… that kind of thing*”) and disagreements with the provider's recommendations for treatment (e.g., “*Yeah, the negative was that they did not care for his arm at all*”). Typical reports from parents also include information about both positive and negative interactions with past providers.

Parent reports that were categorized as variant include providers not believing the child or not taking the pain seriously (e.g., “*Sometimes I get the feeling that some of the doctors might not believe her*”), providers having a general lack of expertise in treating pediatric pain, and statements by parents about how they think the provider should share some information with the parent without the child in the room. Finally, variant reports also describe difficulty with inadequate health insurance coverage (e.g., “*And you gotta accept that person's opinion because that's all your insurance is going to pay for is that one person*”).

### 3.2. Parents Attitudes toward the Experience and Treatment of Pediatric Pain

#### 3.2.1. Parent Attitudes toward Pain

The domain Parent Attitudes toward Pain includes any attitude expressed by the parent regarding pain, pain management, and related areas of functioning. This domain includes three typical (Medications, Stressful Situations, and Negative Emotions) and six variant (Adherence, Eliminate Pain, Functioning, Helplessness, Treatment, and Unknown) core ideas. Typical parent interviews refer to parents' aversive attitudes towards managing pediatric pain with medications (e.g., “*I do not want him takin' a whole lot of pills at his age*;” “*I truly believe that, you know, alternative medicine can certainly work*”), descriptions of stressful situations surrounding child's pain and parents' negative emotions about child's pain, (e.g., “*I'm like basically on the verge of crying because I hate seeing her in pain*”). Variant topics include the importance of adherence to the team's treatment plan to alleviate pain, the parents' desire to eliminate their child's pain (e.g., “*I just want him to stop being in pain, I just want him to turn back into the kid he was in, he was before all this*…”), the need for improving the child's level of functioning while having pain (e.g., “*She's just lying in bed… we've got to do something to get her moving*”), the parents' feeling that they cannot do anything to alleviate their child's pain (e.g., “*They are the people who can… I cannot write a prescription. I cannot do anything*”), the parents' attitudes towards their child's treatment for pain, and the frustrating experience of having unknowns about treatment and diagnosis of child's pain (e.g., “*I'm most concerned about… number one, getting a diagnosis*”).

#### 3.2.2. Parent's Preconceptions of Appointment

The domain Parent's Preconceptions of Appointment includes information about parent's emotions and thoughts about going to the clinic prior to the clinic visit. This domain includes one general (Hope), three typical (Mixed Expectations, No Knowledge, and No Expectations), and two variant (Low Expectations and Meeting with Team) core ideas. Hope is a general theme across interviews; eight of the parents interviewed expressed hope for treatment's effectiveness in decreasing the child's pain (e.g., “*You hope that there is something there that can help*;” “*I guess my strongest feeling was hopeful, just really hopeful that we would get answers/results out of it*”). In typical interviews, parents expressed not knowing what would occur (e.g., “*I did not know… I had never been to a pain clinic before. I did not know what they would do*”), mixed expectations (e.g., “*… but we've had mixed results from other treatments so in the back of your head your thing is this going to be good or another 1.5 hour visit that will be a waste of time*”), or no expectations about the appointment at the pain clinic. Variant interviews include reports of low expectations about the clinic appointment and discussions about the clinic appointment being with a team of providers rather than a single provider.

### 3.3. Families' Perceptions of the Multidisciplinary Clinic

#### 3.3.1. Parent Attitudes toward Multidisciplinary Team

The domain Parent Attitudes toward Multidisciplinary Team includes statements regarding parents' positive or negative thoughts and emotions regarding the general experience with the multidisciplinary team and approach. This domain includes one general (Positive Shift in Emotions), 13 typical (Child Involvement, Collaboration, Commitment, Expectations Exceeded or Met, Expertise, Faith and Hope, Neutral Appointments, Neutral Team, Neutral Treatment, Organization of Team, Privacy, Thoroughness, and Warmth), and three variant (Dislike of Retelling History, Negative and Neutral Emotion) core ideas. Generally, parents described feeling more positively about attending the clinic throughout and after the appointment (e.g., “*Yeah, as the meeting started going on, I started feeling more comfortable, more positive, more open; you know more trusting*”).

Typically, parents reported positively about the organization of team members and team preparation for the appointment, the warmth of the team members (e.g., “*The whole group and made him feel comfortable, made us both feel comfortable when we first got there. So I really liked that*”), the inclusion of the child in appointment discussions and also having time to talk about the child with team members without the child (e.g., “*There were maybe some psychological aspects to it, that I really wanted to explore and, and talk about without him being there*”). Also regarding the multidisciplinary approach of the clinic, parents typically described the team's thoroughness and breadth in addressing all aspects of child's pain (e.g., “*There are other aspects of pain that have a spiritual side and a mental component and lifestyle components, and I like that he addressed different sides. He did not call it that, I said it. It was nice*”), the collaboration or “think tank” style of the team (e.g., “*I like the idea of all of them talking to each other and brainstorming to try to come up with different solutions to the problem*”), the potentially long-term commitment and efforts of team members to alleviate the child's pain, and the expertise or experience of the team members in treating pediatric pain. Parents also typically reported their expectations regarding the appointment and/or care being exceeded or met by the team, and expressed their faith or hope in the team's ability to alleviate the child's pain.

There were also neutral attitudes regarding the multidisciplinary team expressed in interviews. There were typical attitudes or evaluations regarding the appointment, multidisciplinary team and treatment at the clinic, which were neither positive nor negative. For example, statements such as “it was fine” or parents noting having no positive or negative opinion or attitude were included as neutral.

Core ideas classified as variant include statements about the family being appreciative of not having to retell the medical history repeatedly due to the multidisciplinary approach of the clinic, some negative interactions with the team (e.g., “*And they [treatment team] came in there like, and did not really have much information. Point blank [they] said that she was the first child that they were dealing with [her needs].*”) and emotions regarding the team members which were neither positive nor negative.

#### 3.3.2. Patient's Perceptions of the Multidisciplinary Approach

The domain Child's Attitude toward Multidisciplinary Team was applied to parents' recounting of child's thoughts and emotions regarding the experience of the multidisciplinary approach. This domain includes two typical (Negative Attitude and Team Positive) and two variant (Treatment Neutral and Treatment Positive) core ideas. Typically parents reported statements made by the child indicating positive attitudes towards the team (e.g., “*He just say they was nice. He say, “Mama they was nice or something*.”), while others made statements indicating negative attitudes towards the team, treatment, and appointments. Variant reports indicate neutral or positive attitudes towards the treatment, respectively.

#### 3.3.3. Parent-Clinic Provider Relationship

The domain Parent-Clinic Provider Relationship describes parents' contact and rapport with individual providers at the pain clinic. This domain includes two typical (Expertise and Warmth) and two variant (Eliminate Pain and Individual Care) core ideas. Typical interviews include parents' descriptions of the provider as knowledgeable and giving good explanations to the family (e.g., “*He made a lot of sense, because I knew what he was talking about with nerves, because nerves try to re-grow and I was impressed that he even knew that*”) and describing the provider as nice, personable, and approachable (e.g., “*I guess I would have to say the therapist… I met with her first and she really hit right on point with some of the emotional things that's going on with my son. It's like she knew him before she even met him*”). Variant core ideas include descriptions of the provider's focus on eliminating or ending the child's pain and of the individually focused care of the provider (e.g., “*But, I like the idea of being able to call [the nurse]. [The nurse] says well okay let us do this… you know, let us try this for two more days and then she told me to call her back today in fact to give her an update*”).

### 3.4. Concerns Regarding Treatment from the Multidisciplinary Team

#### 3.4.1. Patient's Noncompliance

Patient's Non-Compliance is the domain including information regarding the child's noncompliance with the treatment plan as prescribed by the pain clinic or as implemented by the parents. There are two typical core ideas (General Failure and Medications) and one variant core idea (Attitude) within this domain. Typical reports describe the child's failure of following through with the treatment plan (e.g., “… *the part that's causing the most trouble is the fact that he's not compliant with some things… he does not want to do the things that are going to keep him healthy*”) and the child's desire to not take the medications (e.g., “*I wanted him to start on his medicine yesterday, but he said he want to start on it today*”). A variant theme describes the child's attitude contributing to non-compliance (e.g., “*[The child] tends to give vague answers a lot of time or she'll shrug her shoulders and say I do not know she's at an awkward age and she does not like doctors because she's seen so many of them*”).

#### 3.4.2. Treatment Plan

The domain Treatment Plan includes parents' attitudes, thoughts, and emotions towards the treatment plan given to the child by the pain clinic team. This domain includes six typical (Hope, Medication Positive, Mental Health, Neutral, Openness, and School) and two variant (Not Working and Physical Therapy) core ideas. Typically, parents expressed hope or faith in the treatment plan (e.g., “*She went step-by-step to show me what they would do to correct the problem and she gave us a lot of hope and I'm real confident that he is going to use his arm again*”), positive opinions toward medication as part of the treatment plan, descriptions of the mental health aspects of the treatment plan (e.g., “*Let's fix that. Let's help her with the emotional state of mind to go up, and that will help with the pain in the end. I like that*”), neutral opinions and statements about the treatment plan, willingness in trying the treatment plan (e.g., “*And like I said, we are willing to try… whatever they do, we do*”),and school-related aspects of the treatment plan (e.g., attendance, reintegration, individualized education plans). The two core ideas classified as variant include statements describing the child as not having had significant improvement (e.g., “*Like right now the program has not worked as of yet, but we've been on it a week with the treatment plan*”) and physical therapy being part of the treatment plan.

## 4. Discussion

The results demonstrated a variety of impressions that parents had regarding their intake appointment at a pediatric multidisciplinary pain clinic. The two most common themes mentioned across interview were feeling more positively about attending the clinic after the appointment and hope for treatment's effectiveness in decreasing the child's pain. Based on the parents' descriptions of past treatment for their children's pain, it is possible that the multidisciplinary team and integrated treatment approach offered families new hope for improvement. Most parents had described seeing a variety of medical providers, being in a hospital, and using medication to manage pediatric pain; some parents reported aversive attitudes towards managing pediatric pain with medications.

Alternatively, parents expressed positive reactions toward the medication, mental health, and school reintegration aspects of the treatment plan offered by the multidisciplinary team. Across most interviews, parents mentioned never having had a treatment plan in place for the child's pain before coming to the pain clinic. Parents were willing to try the team's treatment plan and expressed hope that it would work. Possible explanations for parents' positive attitudes towards the treatment plan could be the relationship with the members of the team themselves, or the integration of team members as a whole. Parents may have also been responding positively to the time the team members took to conceptualize the youth's pain, behaviors, and emotions in an integrated fashion, and the stepwise approach taken to create a treatment plan which was provided in written format to the families.

Parents frequently commented on the team member's preparation for the appointment, and the warmth and knowledge of the team members. Parents also mentioned that they liked the “think tank” style of the team. Overall, parents typically reported that their expectations regarding the appointment with the multidisciplinary team were exceeded and expressed their faith or hope in the team's ability to alleviate the child's pain. This high level of faith and hope is assumed to be due to the multidisciplinary treatment approach and high level of team members' expertise treating children with pain. Throughout the evaluation, team members assess child and family beliefs. This careful assessment results in the ability of the team to frame and conceptualize the problem and treatment in a way that matches families unique belief system. This family-centered approach likely conveys a hope and belief in the patient and families ability to address pain more effectively. However, it is currently unclear if parents had the same level of hope with each new provider they encountered during their search for treatment for their child's pain.

Some parents also noted a sense of uncertainty prior to the initial appointment, particularly regarding the structure of the appointment. Specific concerns reported included unknown length of appointment, no knowledge that child would be separated from parent so providers could speak with them separately, and which health care providers would be present at the appointment. Parents may also have expressed uncertainty because of preconceived perceptions that individuals are referred to pain clinics when doctors are giving up on the treatment and no longer looking for a diagnosis for the pain experience. Many parents reported having no specific expectations of the initial appointment, however, these parents sometimes noted that the appointment was not what they expected it to be. Despite receiving information when they schedule and a letter prior to the appointment describing the structure of the appointment and which providers will be attending the appointment, parents may require a more concise and informative method to understand the structure of the appointment, including which health providers are present and the logistics of the appointment, in order to decrease uncertainty regarding the initial appointment. It is also possible that given the unique, multidisciplinary format of the initial evaluation, information about the visit does not match past experiences within the healthcare system and therefore parents do not have a context with which to understand the format.

The results from this study indicate that overall parents were either satisfied or very satisfied with the response they and their children received from the multidisciplinary chronic pain clinic team. This response not only refers to the specific treatment plan given to the family for the child's pain but also the interpersonal interactions with the providers. Parents also noted that they experienced increased satisfaction with the multidisciplinary team as compared to previous health providers. Based on the literature regarding patient satisfaction and treatment adherence, patients are more likely to adhere to treatment plans provided by their health provider when the patients are satisfied with the providers [20–22]. It has been noted that there are three aspects to health care provider communication manners that may influence parents' perceptions of their children's care: information provided, interpersonal manners, and building partnerships with parents/families [23]. Parents in the current study addressed all three of these factors, noting that the treatment team provided them with more information than the parents received from past providers, the treatment team treated the parents and children with respect unlike many past providers, and included both parents and children into much of the treatment plan discussion also unlike many past providers.

This satisfaction with the treatment team can lead directly to increased treatment adherence, as when family members feel a better relationship with their treatment provider, and that the treatment provider acknowledges their concerns [24], and when the family members are partners with their medical providers, they are more likely to adhere to treatment [25]. This indicates that children treated by a multidisciplinary chronic pain team would likely display increased adherence to treatment plans provided given the high levels of satisfaction reported by parents with the treatment they and their children received.

Biopsychosocial assessment and treatment in multidisciplinary chronic pain clinics have become more common for children and adolescents [10]. Treatment at a multidisciplinary treatment center may be a welcome change for these families as treatment for the youth's pain may be offered by multiple providers in one location and often times in one appointment. Overall, families may experience a sense of relief when entering a multidisciplinary clinic specializing in treating children with chronic pain, as some families attend multiple appointments and receive various treatment plans prior to an accurate diagnosis of the child's pain. Disbelief of pain severity is not experienced as often when meeting with multidisciplinary treatment teams [7].

### 4.1. Limitations

 This study provides information regarding parents' perceptions of treatment for various types of chronic pain at a multidisciplinary chronic pain clinic. Given the nature of the qualitative coding method used for this study, the small sample size of local participants does not provide a very heterogeneous sample. However, this small sample allowed for a more in-depth assessment of parents' perceptions of the initial appointment. As well, the demographic information for this sample was not collected at the time of recruitment. Both of these factors make the sample difficult to generalize to all families with chronic pain treated at any multidisciplinary clinic. Given the retrospective nature of data collection, there is a possibility that parents' reports may not be precise regarding their expectations of the clinic prior to the appointment and their initial perceptions of the clinic and appointment. The semistructured nature of the interview protocol also elicited opinions and attitudes regarding certain aspects of the initial appointment, thereby reducing the focus on opinions and attitudes naturally expressed by the parents. Another potential limitation of this study is that researchers recruited parents at the clinic. Though it was clearly explained to parents that the results from this study would not interfere with their child's care at the clinic, there is potential that parents were withholding negative perspectives they had of the clinic and the treatment team.

## 5. Conclusions

Parents generally reported a sense of met or exceeded expectations however, a sense of uncertainty prior to the initial appointment was also reported. Parents noted being uncertain about what the appointment would involve and who would be present at the appointment. It is possible that due to limited use of multidisciplinary teams in health care, parents are not aware of what to expect in a team appointment despite explicit description via letter. It would be beneficial for pediatric multidisciplinary clinics to provide more concrete information regarding the team appointment approach to ensure that parents understand the structure of the multidisciplinary team and treatment approach prior to the initial appointment. This increased information regarding the logistics and structure of the clinic and appointment may ease some of the parents' uncertainty or hesitation prior to the initial appointment. This information can be provided by phone when the appointment is scheduled or by mail prior to the appointment. Parents may report their sense of uncertainty or unease to other medical providers prior to their initial appointment at the multidisciplinary clinic; therefore, it is important to increase other providers' knowledge of the structure and treatment approach of the multidisciplinary pain clinic, perhaps with a focus on explaining the importance of including a mental health provider in the treatment team.

Prior to study, the multidisciplinary team had tailored the initial evaluation process to meet the perceived needs of individuals and families coming to the pain clinic. This study provides insight into families' responses to this unique multidisciplinary approach. This study validates that the team approach focused on empathy, collaboration, treatment planning, and increasing hope in treatment are all valued by parents. The biopsychosocial perspective is perceived by parents as a better fit for treatment of their children's chronic pain than the purely medical perspective many encountered prior to their appointment at the chronic pain clinic. This positive perception of the rationale for treatment and prognosis may in turn increase parents' and patients' interest and investment in the treatment plan provided by the chronic pain clinic treatment team. Future research should explore if the increased hope perceived by parents of the multidisciplinary treatment team using a biopsychosocial approach results in increases to treatment adherence as compared to other approaches (e.g., treatment based solely on the medical perspective). Researchers should also assess how the increased hope and increased treatment adherence influence the treatment's efficacy in reducing the child's pain and increasing their level of functioning. Research focusing on these factors could lead to improved treatments for the children with pediatric chronic pain and their families.

## Appendix

### A. Parent Interview Guide

*Age*.

Race/Ethnicity.

How long has your child been experiencing chronic pain?
What is the experience of patients' parents after the first encounter with the multidisciplinary pain treatment team?
How do their experiences differ from experiences they have had with other clinical care teams?
Expectations and experiences throughout your first meeting.
  1. Please tell me about your thoughts and emotions about your child's first meeting with the multidisciplinary pain clinic team before having the appointment.

Possible Probes.

What were the things that you were most concerned about?
What were the things that you were the most excited for?
Please describe the strongest feelings associated with coming in to meet the pain team for the first time.
  1. Please tell me about some of your expectations of your child's first clinic appointment.

Possible Probes.

Were your expectations mostly positive, negative, or mixed? Could you please describe some of them?
Please tell me about your expectations of a new set of professionals treating your child's pain.
Tell me about how hopeful you were about that the multidisciplinary treatment approach would help your child before coming in for treatment.

Description of How Feelings Changed throughout Session

1. Please tell me about your thoughts and emotions throughout your child's first meeting.

Possible Probes.

Please tell me about how the team was both similar and different to your expectations.
Please tell me about the changes in your emotions, thoughts, and expectations throughout the meeting.
What was the most positive and negative thing that you heard?
If your emotions changed throughout the meeting, please tell me about the most drastic change in your expectations, emotions, and thoughts.
Please tell me about how hopeful you felt as you met the treatment team and heard about the treatment process.
Please tell me about your impressions of the treatment team.
  (2) Please tell me about the elements of your initial appointment that were the most noticeable to you.

Possible Probes.

Please tell me about your experience meeting with the team as a group.
Please tell me about your experience and feelings/thoughts about different members of the family meeting with different team members.
Please tell me about your experience and feelings/thoughts about the treatment plan.
Please tell me about the most noticeable or distinct part of the meeting.
Thoughts about the treatment after leaving the session.
  (3) How was the meeting similar to and/or different from what you wanted, and also what you expected?

Possible Probes.

Please tell me your feeling and thoughts about how your expectations were met or not met during the session.
Please tell me about your level of hopefulness after leaving the first session. If this changed, please discuss your experience of having your level of hope change.
Please tell me about your reactions to both what and how the team members discussed your pain in your first clinic meeting.
Please tell me about the most positive and negative experiences you had during your first visit.
Please tell me about how much faith you have in this current treatment, and what contributed to your feelings about this.
Please discuss some of the characteristics about the treatment staff that you believe will help your child deal with his/her pain, and what about the treatment staff might be the most challenging in helping your child deal with his/her pain?
  (4) Please tell me about your family's experience after leaving the session.

Possible Probes.

Did the session prompt any changes in your family's behavior, conversations, or thoughts regarding your child's pain? Please tell me about those.

Contrast with Previous Medical Care.

(1). Please tell me about how well you felt previous medical teams dealt with your child's pain

Possible Probes.

Tell me about the best and worst experience you had with a member of a different treatment provider or team.
What about each of these situations was the most positive and negative part?
What were the best and worst things that you were told from previous providers?
  (2) How were your previous experiences similar to and different from your experience with the multidisciplinary pain clinic team?

Possible Probes.

Please tell me about your thoughts/feelings/reactions to the similarities and differences with previous pain treatment experiences.
Was your level of hopefulness higher, lower, or the same than it was in previous medical situations, and what do you think made this similar or different?
How was the communication with this treatment team both similar and different to your communication with other treatment professionals?
What elements of this treatment do you believe will be helpful to you? How do you see this as both similar to and different from other treatments for your child's chronic pain?
Please tell me what was similar or different about members of this treatment team in comparison to other providers your child has seen.
